# Using 4DCT‐ventilation to characterize lung function changes for pediatric patients getting thoracic radiotherapy

**DOI:** 10.1002/acm2.12397

**Published:** 2018-06-26

**Authors:** Yevgeniy Vinogradskiy, Austin Faught, Richard Castillo, Edward Castillo, Thomas Guerrero, Moyed Miften, Arthur K. Liu

**Affiliations:** ^1^ Department of Radiation Oncology University of Colorado School of Medicine Aurora CO USA; ^2^ Department of Radiation Oncology Emory University Atlanta GA USA; ^3^ Department of Radiation Oncology Beaumont Health System Royal Oak MI USA

**Keywords:** CT ventilation, dose‐–response assessment, lung function imaging, pediatric radiotherapy

## Abstract

**Purpose:**

A form of lung functional imaging has been developed that uses 4DCT data to calculate ventilation (4DCT‐ventilation). Because 4DCTs are acquired as standard‐of‐care to manage breathing motion during radiotherapy, 4DCT‐ventilation provides functional information at no extra dosimetric or monetary cost. 4DCT‐ventilation has yet to be described in children. 4DCT‐ventilation can be used as a tool to help assess post‐treatment lung function and predict for future clinical thoracic toxicities for pediatric patients receiving radiotherapy to the chest. The purpose of this work was to perform a preliminary evaluation of 4DCT‐ventilation‐based lung function changes for pediatric patients receiving radiotherapy to the lungs.

**Methods:**

The study used four patients with pre and postradiotherapy 4DCTs. The 4DCTs, deformable image registration, and a density‐change‐based algorithm were used to compute pre and post‐treatment 4DCT‐ventilation images. The post‐treatment 4DCT‐ventilation images were compared to the pretreatment 4DCT‐ventilation images for a global lung response and for an intrapatient dose–response (providing an assessment for dose‐dependent regional dose–response).

**Results:**

For three of the four patients, a global ventilation decline of 7–37% was observed, while one patient did not demonstrate a global functional decline. Dose–response analysis did not reveal an intrapatient dose–response from 0 to 20 Gy for three patients while one patient demonstrated increased 4DCT‐ventilation decline as a function of increasing lung doses up to 50 Gy.

**Conclusions:**

Compared to adults, pediatric patients have unique lung function, dosimetric, and toxicity profiles. The presented work is the first to evaluate spatial lung function changes in pediatric patients using 4DCT‐ventilation and showed lung function changes for three of the four patients. The early changes demonstrated with lung function imaging warrant further longitudinal work to determine whether the imaging‐based early changes can be predicted for long‐term clinical toxicity.

## 1 INTRODUCTION

A form of functional imaging has been developed that uses 4‐dimensional computed tomography (4DCT) data along with image processing techniques to calculate 4DCT‐based lung ventilation maps.^1^, ^2^, ^3^ 4DCT‐ventilation has been gaining momentum in radiation oncology because 4DCT simulations are frequently acquired as part of the standard treatment planning process; which enables the calculation of 4DCT‐ventilation‐based lung function without burdening the patient with an extra imaging procedure. There have been two clinical applications of 4DCT‐ventilation in radiation oncology: functional radiotherapy (designing radiation treatment plans that minimize dose to functional lung) and thoracic treatment assessment.^4^, ^5^, ^6^, ^7^

One clinical setting where 4DCT‐ventilation has yet to be explored is children receiving radiotherapy to the lungs. Some common indications for radiation to the lungs in pediatric patients include whole lung radiation for metastatic solid tumors, total body irradiation, and focal radiation for primary or metastatic disease in the chest. As with adult patients, pulmonary complications are a serious clinical concern for pediatric patients that get dose to the thorax.^8^

In this work, we evaluate the concept of using 4DCT‐ventilation in the pediatric setting. Thoracic toxicity is an important clinical concern for pediatric patients getting lung radiotherapy. As with adult patients, 4DCT simulations are often used for pediatric patients where breathing motion management is needed. Therefore, acquiring baseline lung function with 4DCT‐ventilation for pediatric patients would still come at minimal monetary, dosimetric, or time cost to the patients. 4DCT‐ventilation can potentially be used to help assess post‐treatment lung function and predict for subsequent long‐term clinical thoracic toxicities. The purpose of this work was to evaluate 4DCT‐ventilation‐based spatial lung function changes for pediatric patients getting thoracic radiotherapy.

## 2 METHODS

### 2.A Study population

**Figure 1:**
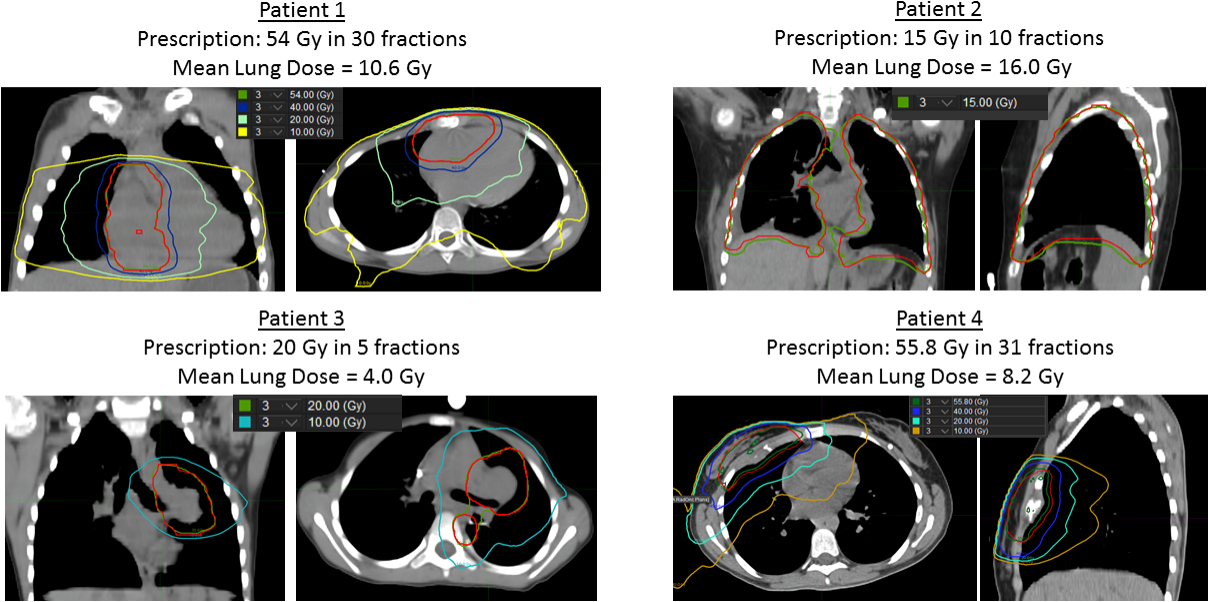
Radiation isodose lines and lung dosimetric data for the four patients used in the study.

Four patients (referred to as Patient 1, Patient 2, Patient 3, and Patient 4) were selected for analysis that had a pretreatment 4DCT acquired and a follow‐up 4DCT available (acquired as part of a simulation for a subsequent treatment). Patient ages at the time of treatment were 10, 11, 6, and 16 years old for patients 1, 2, 3, and 4, respectively. Patient 1 was treated for metastatic cardiac angiosarcoma and received 54 Gy in 30 fractions. Patient 2 received whole lung radiation (15 Gy in 10 fractions) for the treatment of small round cell sarcoma. Patient 3 received treatment to a left hilum PTV lung metastasis (20 Gy in five fractions) and Patient 4 received 55.8 Gy in 31 fractions for the treatment of a Ewing's Sarcoma rib lesion. The isodose profiles and lung dosimetry for each patient are shown in Fig. 1. The times between the pre and post‐treatment 4DCTs were 237, 133, 71, and 233 days for patients 1, 2, 3, and 4, respectively.

### 2.B 4DCT‐ventilation image calculation

**Figure 2:**
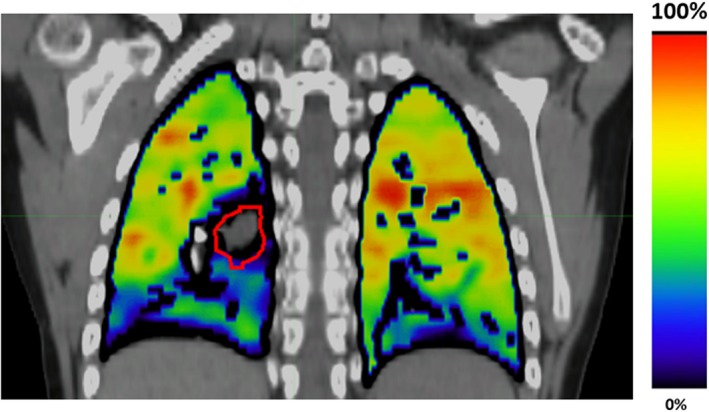
An example of a 4DCT‐ventilation image overlaid with a standard CT. The bright colors represent function lung while the darker tones display areas of lower functioning lung. The displayed patient presents with a mass (outlined in red) that is occluding an airway and is causing a defect in the right lower lobe.

Each patient's pre and post‐treatment 4DCT scan was used to calculate 4DCT‐ventilation maps using previously described methods.^1^, ^2^, ^9^ The lungs are first segmented on the 0% (Inhale) and 50% (Exhale) phases of the 4DCT data set. Deformable image registration is used to register lung voxels from the inhale to the exhale data set.^10^ Once the inhale and exhale voxels were linked, a density‐change‐based equation was applied to calculate ventilation:

(1) $$\frac{V_{\mathrm{in}}-V_{\mathrm{ex}}}{V_{\mathrm{ex}}}=1000\frac{{\text{HU}}_{\mathrm{in}}-{\text{HU}}_{\mathrm{ex}}}{{\text{HU}}_{\mathrm{ex}}\left(1000+{\text{HU}}_{\mathrm{in}}\right)}$$

where *V*_in_ and *V*_ex_ are the inhale and exhale volumes and HU_in_ and HU_ex_ are the inhale and exhale Hounsfield units of the individual lung voxels. Eq. (1) is then applied on a voxel‐by‐voxel basis to produce a 3D map of ventilation. Eq. (1) results in a unitless ratio of the amount of change in air content from inhale to exhale.^2^, ^11^ For display and evaluation purposes, we multiplied all raw ventilation values by 100. An example of a ventilation image calculated for Patient 3 is shown in Fig. 2. In order to facilitate a 4DCT‐ventilation comparison at different time points, the 4DCT‐ventilation images were registered (using MIM Vista Version 6.7 [Cleveland, OH]) and normalized.^12^, ^13^, ^14^

### 2.C Evaluation of radiation‐induced 4DCT‐ventilation changes

For each patient, we compared the post‐treatment 4DCT‐ventilation image to the pretreatment 4DCT‐ventilation image. The comparison was done in two ways: (1) Total ventilation change in the entire lung, and (2) intrapatient, regional ventilation dose–response. To evaluate total ventilation change, the average ventilation values in the pre and post‐treatment 4DCT‐ventilation image were calculated. We then assessed the difference between the average ventilation in the post‐treatment ventilation image against the average ventilation in the pretreatment ventilation image.

An intrapatient dose–response analysis was done to evaluate 4DCT‐ventilation changes as a function of delivered dose. The intrapatient dose–response enabled a direct assessment of whether there was a differential ventilation response as a function of delivered dose. The intrapatient dose–response was first assessed by evaluating ventilation changes within and outside of the 10 and 20 Gy isodose lines (three of the four patients used for dose–response evaluation did not receive lung doses higher than 20 Gy to the lungs). In addition, we created dose–response curves by binning the ventilation values in dose bins of 5 Gy and calculating the change in ventilation for each dose bin. For Patients 1 and 3, the dose bins were evaluated up to 20 Gy while for Patient 4 the dose bins were evaluated to 50 Gy. Patient 2 received a homogenous dose of 15 Gy and was therefore only evaluated in the 15 Gy dose bin.

## 3 RESULTS

**Figure 3:**
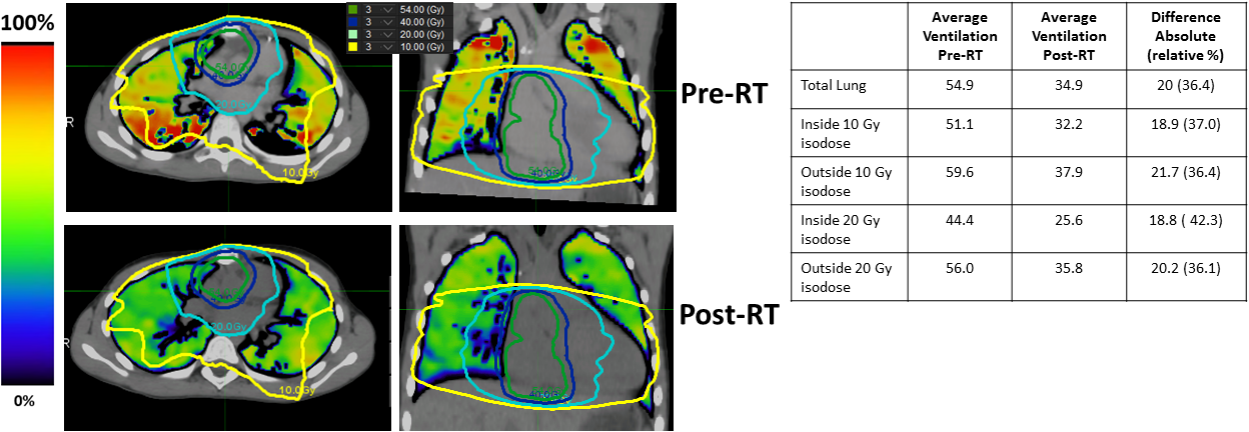
Pre and post‐treatment 4DCT‐ventilation images for Patient 1. The treatment isodose lines are overlaid over both of the ventilation images. The accompanying quantitative ventilation results for Patient 1 are presented in the table. The presented patient displays a global ventilation decline with no differential decrease in functional in the treated region.

The pre and post‐treatment ventilation images overlaid with the dose distribution for Patient 1 are shown in Fig. 3. The ventilation images qualitatively illustrate an overall decline in lung function from pre to post‐treatment. Visually, there are no differential decreases in lung function in regions that are encompassed by the 10 and 20 Gy isodose lines compared to lung regions that were outside of those isodoses. The quantitative results echo the visual observations. The average lung ventilation of the pretreatment image was 54.9 and the average ventilation of the post‐treatment scan was 34.9 for a difference of 20.0 (36.4% relative decline). The decrease in ventilation did not vary according to isodose lines. For example, the decrease in ventilation from pre to post‐treatment inside the 20 Gy contour was 18.8, while the decrease outside the 20 Gy contour was 20.2.

Patient 2 (who received whole lung irradiation) exhibited a global decline in lung function. The pretreatment average ventilation was 48.0 and the post‐treatment ventilation value was 29.9 for a ventilation decline of 18.1 (37.7% relative). No isodose analysis was done for Patient 2 because the patient received a uniform lung dose. Patient 3 exhibited no global ventilation differences between the pre and post‐treatment ventilation images. The average pretreatment ventilation value was 45.4 and the average post‐treatment value was 46.4 for an increase in ventilation of 1.0.

**Figure 4:**
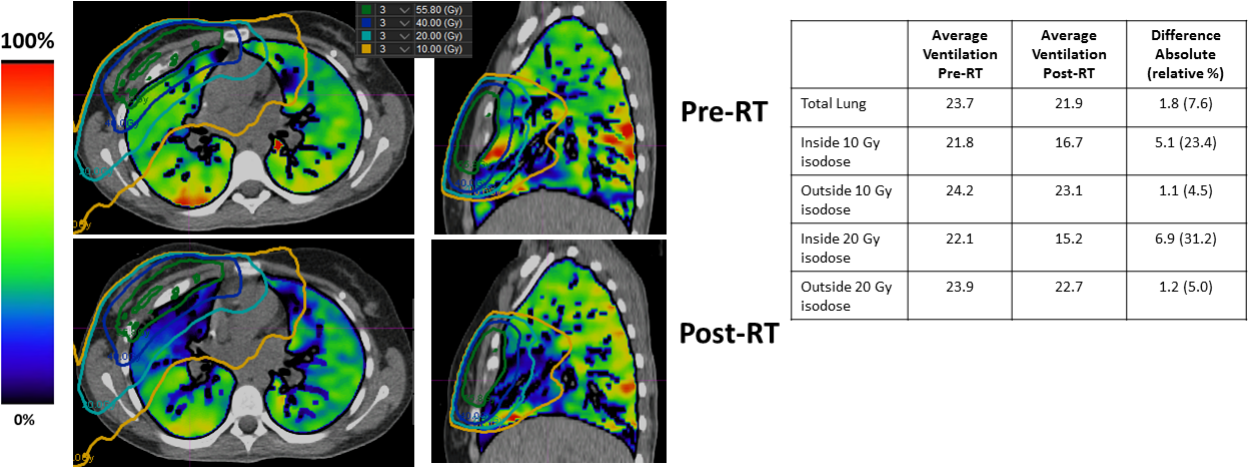
Pre and post‐treatment 4DCT‐ventilation images for Patient 4. The treatment isodose lines are overlaid over both of the ventilation images. The accompanying quantitative ventilation results for Patient 4 are presented in the table. The presented patient displays a 4DCT‐ventilation‐based functional decline in the treated lung regions.

The pre and post‐treatment ventilation images overlaid with the dose distribution for Patient 4 are shown in Fig. 4. The ventilation images demonstrate a qualitative functional decline in the treated lung regions. The quantitative results echo the qualitative functional decline observations. For example, the ventilation values inside the 20 Gy isodose demonstrated a 31.2% relative decrease in ventilation, while the ventilation values outside of the 20 Gy isodose line displayed a 5.0% decrease in ventilation.

**Figure 5:**
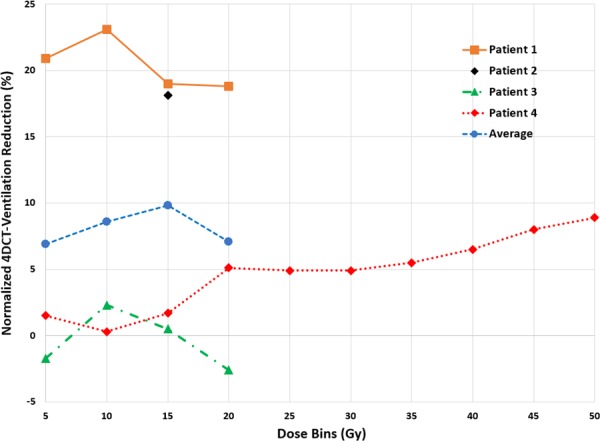
Dose–response curves averaged over the entire population and for each individual patient. Data points represent the reduction in normalized 4DCT‐ventilation value for each dose bin.

Dose–response curves for all four patients and averaged over the entire cohort are shown in Fig. 5. The individual dose–response curves echo the results for the individual patients; Patients 1 and 2 demonstrated global ventilation decline; Patient 3 did not demonstrate overall ventilation reduction, and Patient 4 presented with a clear regional dose–response relationship (increased 4DCT‐ventilation decline as a function of increasing dose).

## 4 DISCUSSION

The results point to two distinct observations. The first observation is that in three out of four patients we demonstrated a global reduction in lung function, while in one patient there was no change in pre to post‐treatment ventilation. The second finding was that we did not observe any regional reduction in ventilation as a function of isodose level for three out of the four patients, while for one patient there was a clear dose–response with decreasing 4DCT‐ventilation values with increasing lung dose. The findings warrant discussion from several different perspectives. From the perspective of pediatric thoracic toxicity, it was surprising that an imaging‐based change in lung function was evident within 237, 133, and 233 days for patients 1, 2, and 4, respectively. Radiation pneumonitis can manifest 3–12 months after treatment^15^ while clinical and PFT‐based thoracic toxicity typically manifests 5–10 years after treatment for pediatric patients.^16^ Our study is one of the first to present lung function imaging‐based changes for pediatric patients. The early changes demonstrated with lung function imaging warrant further longitudinal work to determine whether the imaging‐based early changes can be a predictor for ensuing clinical problems years after radiotherapy.

Our data indicated mixed dose–response results. Two of the patients demonstrated a global dose–response, one patient did not show any decline in spatial function, and one patient showed both a global and dose‐dependent dose–response (greater ventilation reduction with increasing dose). There have been several spatial lung function dose–response studies in the adult literature.^4^, ^17^, ^18^, ^19^, ^20^, ^21^, ^22^ In general, the pediatric data we present echo the adult literature, in that the lung functional response can be complex, as the radiation damage is juxtaposed with potential improvement from treatment of the lung disease. There are several possible explanations for the mixed results. While adult studies generally report an overall dose–response, the changes in the 0–20 Gy dose range have either been negative or inconclusive.^4^, ^17^, ^18^, ^19^, ^20^, ^21^, ^22^ One possibility is that we were not able to observe an intrapatient dose–response for three patients because the lung doses delivered in our study population did not exceed 20 Gy. Patient 4 received lung doses greater than 20 Gy and exhibited a clear dose–response. Another possible explanation is mean lung dose (MLD); the two patients that demonstrated an overall decline in ventilation had MLDs of 10.6 and 16 Gy, the patient that demonstrated an intrapatient dose–response had a MLD of 8.2 Gy, while the patient that did not show a dose–response had a MLD of 3.9 Gy.

The presented work is one of the first studies to evaluate spatial lung function changes in pediatric patients getting radiotherapy to the thorax. The work should be taken in context as a proof of principle study; no formal statistics were presented and there remains a large uncertainty with four patients and long intervals between treatment and the follow‐up 4DCT‐ventilation imaging. However, despite the small sample size, our study was able to demonstrate varying individual imaging‐based thoracic changes which echoes the results presented in the adult literature.^4^, ^17^ We believe our study presents an exciting proof of concept use of 4DCT‐ventilation to track early functional changes in pediatric patients getting thoracic radiotherapy. A prospective clinical trial collecting pre and postradiotherapy 4DCTs can provide valuable data to evaluate imaging‐based changes for pediatric patients, which can in turn be potentially used to predict future clinical toxicity.

## CONFLICT OF INTEREST

This work was partially funded by NCI/NIH grant R01CA200817 (YV, AF, MM, EC, RC, TG). The authors have no other conflict of interest to disclose.
